# The Value of Web-Based Patient Education Materials on Transarterial Chemoembolization: Systematic Review

**DOI:** 10.2196/25357

**Published:** 2021-05-07

**Authors:** Georgios Antonios Sideris, Aikaterini-Themis Vyllioti, Danai Dima, Michael Chill, Njogu Njuguna

**Affiliations:** 1 Department of Radiology Baystate Medical Center University of Massachusetts Medical School Springfield, MA United States; 2 School of Life Sciences Technical University of Munich Munich Germany; 3 Department of Medicine Tufts Medical Center Boston, MA United States

**Keywords:** transarterial chemoembolization, interventional radiology, interventional oncology, liver cancer, hepatocellular carcinoma, internet, patient education, systematic review

## Abstract

**Background:**

Thousands of web searches are performed related to transarterial chemoembolization (TACE), given its palliative role in the treatment of liver cancer.

**Objective:**

This study aims to assess the reliability, quality, completeness, readability, understandability, and actionability of websites that provide information on TACE for patients.

**Methods:**

The five most popular keywords pertaining to TACE were searched on Google, Yahoo, and Bing. General website characteristics and the presence of Health On the Net Foundation code certification were documented. Website assessment was performed using the following scores: DISCERN, Journal of the American Medical Association, Flesch-Kincaid Grade Level, Flesch Reading Ease Score, and the Patient Education Materials Assessment Tool. A novel TACE content score was generated to evaluate website completeness.

**Results:**

The search yielded 3750 websites. In total, 81 website entities belonging to 78 website domains met the inclusion criteria. A medical disclaimer was not provided on 28% (22/78) of website domains. Health On the Net code certification was present on 12% (9/78) of website domains. Authorship was absent on 88% (71/81) of websites, and sources were absent on 83% (67/81) of websites. The date of publication or of the last update was not listed on 58% (47/81) of websites. The median DISCERN score was 47.0 (IQR 40.5-54.0). The median TACE content score was 35 (IQR 27-43). The median readability grade level was in the 11th grade. Overall, 61% (49/81) and 16% (13/81) of websites were deemed understandable and actionable, respectively. Not-for-profit websites fared significantly better on the Journal of the American Medical Association, DISCERN, and TACE content scores.

**Conclusions:**

The content referring to TACE that is currently available on the web is unreliable, incomplete, difficult to read, understandable but not actionable, and characterized by low overall quality. Websites need to revise their content to optimally educate consumers and support shared decision-making.

**Trial Registration:**

PROSPERO International Prospective Register of Systematic Reviews CRD42020202747; https://www.crd.york.ac.uk/prospero/display_record.php?ID=CRD42020202747

## Introduction

### Background

The World Wide Web has greatly facilitated access to medical knowledge for consumers. Nowadays, 6 to 7 of 10 internet users browse the web in search of health-related answers [1,2]. In fact, consumers are four times more likely to turn to the internet first rather than to a physician [3]. Although most users still believe that physicians are the most trustworthy information source, more than half shape their health-related decisions based on information they obtain from the web and may consequently decide against visiting a medical professional [1-4]. However, the quality of websites is often questionable. Websites may contain distracting information and incomprehensible content and may not meet the standards to facilitate medical decision-making [5-9].

Health literacy (defined as the ability to read, understand, and act on health-related information) is a major determinant of the way people process the information they obtain from the web [10,11]. Older people or those with a low educational level tend to have poor health literacy; practice ineffective ways of web searching; and are more vulnerable to physical, emotional, or financial harm caused by inaccurate information [12-14]. As approximately 36% of adults in the United States lack adequate health literacy, the need for reliable and comprehensible websites has become more critical [11,15].

Patients may be skeptical and nervous when they are referred for a procedure they are unfamiliar with and may lack the capacity to fully process the educational materials they are provided [16]. This holds true for interventional radiology (IR) procedures that, despite their multiple applications and benefits, are not widely known to the general public. Although minimally invasive procedures are generally preferred among patients, there is still a considerable lack of awareness of procedures performed by interventional radiologists [17,18].

One of the most widely used procedures in the armamentarium of interventional oncology is transarterial chemoembolization (TACE), which is recommended as the standard of care for select cases of primary or metastatic liver tumors [19]. Web-based content referring to TACE can be found on a wide range of websites (eg, scientific journals, patient blogs, and commercial websites), most of which target medical professionals rather than the average reader. The availability of high-quality, consumer-friendly websites is essential to ascertain that patients can accurately self-educate and make informed decisions. Improved patient education may also have clinical benefits, as it has been linked to more favorable outcomes, such as lower rates of postchemoembolization pain [20].

Studies evaluating websites that provide patient information on IR procedures have been published previously. McEnteggart et al [21] assessed the readability of websites discussing 7 IR procedures (central venous catheter placement, vertebroplasty, varicocele embolization, deep vein thrombosis treatment, transjugular intrahepatic portosystemic shunt, uterine artery embolization, and peripheral artery angioplasty) and found their readability to be below the recommended grade level. Murray et al [22], Alderson et al [23], and Lee et al [24] evaluated the quality and readability of websites referring to uterine artery, varicocele, and pelvic vein embolization, respectively. Website quality was found to be fair, and readability was suboptimal. However, to date, no study has evaluated web-based patient education resources referring to TACE.

### Objective

The aim of this study is to evaluate the reliability, quality, readability, and completeness (using a novel content score) of websites that provide patient information about TACE.

## Methods

### Overview

A protocol delineating the objectives of the study, the outcomes of interest, and assessment criteria was registered with the International Prospective Register of Systematic Reviews (identification number CRD42020202747). This systematic review was performed according to the PRISMA (Preferred Reporting Items for Systematic Reviews and Meta-Analyses) statement [25].

### Study Design

A keyword analytic tool named *Keywords Everywhere* was used to identify the most common keywords pertaining to TACE that are used in web searches globally [26]. The five keywords that were isolated, followed by their respective search volume (average number of searches performed per month over the last 12 months), were as follows: *tace* (40,500), *tace procedure* (6600), *chemoembolization* (2400), *tace in hcc* (1300), and *transarterial chemoembolization* (1300).

The three most popular search engines based on global traffic rankings were selected: Google, Yahoo, and Bing [27]. The website search was performed on May 24, 2020. Web browser cookies and search history were erased so that the search was not influenced by the reviewer’s prior searches. Geolocation was turned off before the search to eliminate any geographical bias. The first 250 results of the five keyword searches on each of the three search engines were downloaded on a Microsoft Excel spreadsheet. Duplicate websites were initially eliminated by the *Remove duplicates* tab in the Excel sheet. As URL addresses with minor alterations (eg, *http* vs *https*) can redirect to the same website, all the remaining website links were opened by one reviewer (GAS), who manually removed the rest of the duplicates. Inaccessible websites or websites with password-restricted access were excluded.

After removal of duplicate and inaccessible links, websites were excluded if they were (1) not in English, (2) irrelevant, (3) discussing TACE using less than 300 words (similar to the study by Hirsch et al [28]), (4) clearly addressing a scientific audience (eg, journal articles, medical newsletters, and treatment guidelines), (5) containing only medical education materials (eg, lecture slides and e-books), (6) providing only discharge instructions, (7) containing only audiovisual material (video), and (8) describing personal experiences of patients (eg, blogs, diaries, and commentaries). Websites discussing TACE with drug-eluting beads or embolization therapy for liver cancer were considered relevant. Eligible websites that directed to a PDF file were also included.

Webpages that belonged to the same domain and served as a succession of one another were evaluated as a website entity. Webpages that belonged to the same domain but served as an independent and stand-alone resource were evaluated separately. Website screening and extraction of website characteristics were performed by one reviewer (GAS). Comprehensive website assessment was performed by 2 medical doctors (GAS and ATV). To limit bias, only one of the 2 reviewers had experience with TACE (GAS). Both reviewers worked independently on a predefined Excel spreadsheet. Discrepancies were resolved through discussion.

### Website Characteristics

Websites were categorized based on website owners into four categories: nonacademic hospitals (eg, community health care institutions), academic hospitals (eg, university health care institutions), not-for-profit organizations (eg, governmental or nongovernmental organizations and medical societies), and for-profit organizations (eg, private medical groups and commercial companies). The website owner, country of origin, date of creation, and date of the last update were extracted. The presence of a privacy statement and medical disclaimer and the number of images, videos, and advertisements were documented. The word count of each website was measured via a web browser extension named *Word Counter Plus* [29]. Only the words in the main text contributed to the total word count, whereas the text on the margins of the webpage, the contact information, and the references were disregarded. Websites with supplemental video content were excluded from the word count analysis. However, information from the videos was considered when evaluating the content of a website.

### Health On the Net Foundation Code

The Health On the Net (HON) Foundation Code of Conduct is a certification provided by a board of experts (HON Foundation) to websites containing objective and transparent medical information [30]. Websites should adhere to the following eight principles: (1) authority (content is written only by medical professionals), (2) complementarity (information supports and does not replace the physician-patient relationship), (3) confidentiality (readers’ privacy is protected), (4) attribution (sources of information are provided), (5) justifiability (claims are balanced and objective), (6) transparency (contact details of authors are provided), (7) financial disclosure (sources of funding are provided), and (8) advertising (advertised and editorial content are clearly distinguished). A browser extension named *HONcode Toolbar* was used to identify the websites that carried the HON code badge [31].

### Website Assessment Tools

#### Journal of the American Medical Association Score

The Journal of the American Medical Association (JAMA) score was generated to assess the reliability of health-related websites [32]. It comprises four benchmarks: (1) authorship (name, credentials, and affiliations of authors), (2) attribution (references and copyright), (3) currency (creation and review date), and (4) disclosure (ownership, sponsorship, advertising, underwriting, commercial funding arrangements or support, and conflict of interest). The total JAMA score ranges from 0 to 4. Points are awarded based on whether the subdivisions of each benchmark are addressed. Websites mentioning an editorial board for the entire website but not specifically for the TACE-related page were not given credits for authorship.

#### DISCERN Instrument

The DISCERN instrument has been widely used to evaluate the quality of written health information (Multimedia Appendix 1) [33]. It consists of 16 questions, each receiving points from 1 (definitive *no*) to 5 (definitive *yes*). Questions 1-8 assess the reliability of the material, questions 9-15 assess the quality of the content regarding treatment choices, and question 16 is a rating of the overall quality of the publication. To limit subjectivity between the 2 reviewers, the grading system for each question was standardized in advance, based on the DISCERN manual. The total DISCERN score spans between 16 and 80 and breaks down as excellent (68-80), good (55-67), fair (42-54), poor (29-41), and very poor (16-28).

#### TACE Content Score

To evaluate the completeness of the information provided by websites, a novel scoring system was created based on the 2017 Society of Interventional Radiology Quality Improvement guidelines [34] and on our expert opinion (Multimedia Appendix 2). Our TACE content score consists of 35 key points that fall under the following categories: (1) background, (2) indications, (3) contraindications, (4) benefits, (5) preoperative considerations, (6) procedure description, (7) postoperative considerations, (8) additional treatments, and (9) risks. The key points were selected based on what information is expected to be found in materials that provide information to patients. Technical aspects, such as nomenclature or size of chemoembolic agents, were not considered relevant for patients and were therefore not included in our scoring system. Each key point was awarded 2 points for full mention, 1 point for partial mention, and 0 points for no mention. Total TACE content scores are hinged between 0 and 70.

#### Flesch Reading Ease Score and Flesch-Kincaid Grade Level

The Flesch Reading Ease Score (FRES) and Flesch-Kincaid Grade Level (FKGL) are mathematical formulas that take into account the number of words per sentence and the number of syllables per word to quantify the readability of written materials [35]. The FRES measures the complexity of the text and corresponds to the writing style difficulty proposed by the US Department of Health and Human Sciences. The FKGL corresponds to the grade level that the reader must have to comprehend the text. Although the two scores consist of the same core metrics, they correlate inversely, so a website with a higher FRES would have a lower FKGL. Formulas such as the Gunning Fox Index that take into account the total number of complex words (ie, words that contain more than three syllables) were not preferred in our study, as many medical terms (including the word *chemoembolization*) contain more than three syllables. FRES and FKGL indexes were used instead, as they are the most widely used and do not solely weigh polysyllabic words.

Text from each webpage was copied and pasted on a free web-based readability checker named *Readability Formulas* [36]. The selection of words for readability assessment was the same as the aforementioned selection of words for the calculation of word count. The two scores were calculated after text formatting (addition of full stops when absent, removal of references and hyperlinks, removal of bullets, and addition of commas when the listed items were single words). Websites with video content were excluded from readability analysis.

The average reading level of the US population is eighth grade; therefore, it has been suggested that website content should be written at the 6th grade level or lower [37].

#### Patient Education Materials Assessment Tool

The Patient Education Materials Assessment Tool (PEMAT) was developed by the Agency for Healthcare Research and Quality of the US Department of Health and Human Sciences to evaluate the understandability and actionability of patient education materials (printable or audiovisual) [38]. Understandability refers to the ability of the material to be understood by readers of varying levels of literacy, whereas actionability refers to the extent to which the material points out the potential actions that readers must take. Overall, PEMAT consists of 19 items measuring understandability and 7 items measuring actionability. Each item is scored as 0 (*disagree*), 1 (*agree*), or NA (*not applicable*) when appropriate. The sum of all awarded points gets divided by the number of total possible points. The quotient multiplied by 100 gives the final PEMAT score for each subdivision. Materials with scores above 70% are considered adequately understandable and actionable [38].

### Statistical Analysis

Medians and interquartile ranges were calculated for continuous variables, whereas frequencies and percentages were calculated for categorical variables. The chi-square test was used to compare categorical variables among the website categories. Continuous variables were compared among website categories using one-way analysis of variance and Kruskal-Wallis test. Correlation between continuous variables was examined using Pearson or Spearman rank correlation coefficients. Statistical significance was set at *P*<.05. Analyses were performed using SPSS software, version 20.0 (IBM Corporation).

## Results

### Search Results

**Figure 1:**
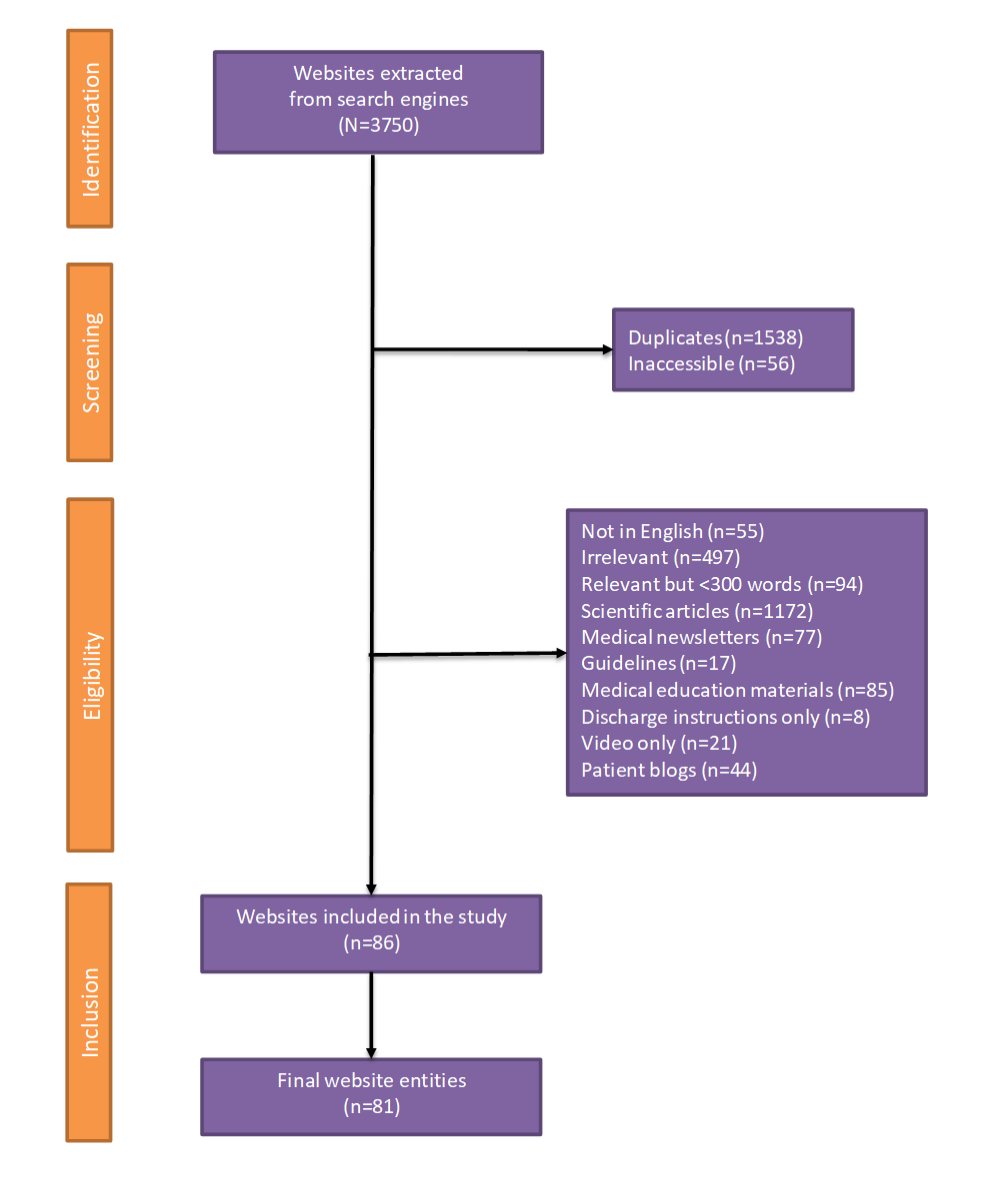
PRISMA (Preferred Reporting Items for Systematic Reviews and Meta-Analysis) flow diagram.

A total of 3750 websites were extracted from the three search engines. Overall, 86 URLs belonging to 78 unique website domains met the inclusion criteria (Multimedia Appendix 3). After grouping the URLs with split chemoembolization content, 81 website entities were evaluated (Figure 1).

### Website Characteristics

The included websites originated from 11 different countries, 62% (50/81) of which were from North America. Of the 81 websites, 15 (19%) belonged to nonacademic hospitals, 29 (36%) belonged to academic hospitals, 21 (26%) belonged to not-for-profit organizations, and 16 (20%) belonged to for-profit organizations.

A privacy statement was provided on 86% (70/78) of website domains. The presence of a privacy statement did not vary by website category. A medical disclaimer was present on 72% (56/78) of website domains. Not-for-profit websites were significantly associated with the presence of a medical disclaimer (*χ*^2^_3_=2.8; *P*=.005), whereas for-profit websites were significantly associated with the absence of a medical disclaimer (*χ*^2^_3_=3.7; *P*<.001).

No illustrations or videos were used on 56% (45/81) of the websites. Of 81 websites, 21 (26%) used only one image and 13 (16%) used more than one image, whereas supplemental videos were used in 5 (6%) websites. Advertisements were displayed on 6% (5/81) of the websites, most of which (4/5, 80%) were in the for-profit category.

The median word count was 765 words (IQR 518.3-1152.3; Table 1). Not-for-profit websites were associated with a significantly higher word count than nonacademic hospitals and for-profit websites (*P*=.04 and *P*=.01, respectively). There was a positive correlation between word count and total JAMA score (*r*_s_=+0.463; *P*<.001), total DISCERN score (*r*_s_=+0.786; *P*<.001), total TACE content score (*r*=+0.665; *P*<.001), and PEMAT actionability score (*r*_s_=+0.548; *P*<.001).

**Table 1:** Assessment results per website category and overall.

Assessment tools	Nonacademic hospitals (n=15), median (IQR)	Academic hospitals (n=29), median (IQR)	Not-for-profit organizations (n=21), median (IQR)	For-profit organizations (n=16), median (IQR)	Total (N=81), median (IQR)
Word count	678 (435.3-873.8)	718 (501.5-1268.8)	1091 (792.5-1785.5)	544 (403.0-763.0)	765 (518.3-1152.3)
Journal of the American Medical Association	1.17 (0.83-1.17)	1.17 (0.83-1.50)	1.67 (1.33-2.25)	1.25 (0.83-1.67)	1.33 (0.83-1.75)
DISCERN	45.0 (37.0-52.0)	47.0 (37.5-54.0)	55.0 (49.0-62.0)	43.5 (34.0-46.0)	47.0 (40.5-54.0)
Transarterial chemoembolization content score	31.0 (29.0-43.0)	34.0 (26.5-41.5)	42.0 (35.0-46.0)	31.0 (23.3-33.8)	35.0 (27.0-43.0)
Flesch Reading Ease Score	43.1 (31.1-52.8)	47.9 (36.1-62.6)	48.9 (45.3-58.4)	41.9 (31.6-48.4)	47.0 (38.7-57.8)
Flesch-Kincaid Grade Level	11.2 (10.0-12.9)	10.8 (8.3-13.4)	10.6 (8.7-11.8)	11.9 (10.4-12.7)	11.2 (8.9-12.6)
PEMAT^a^ understandability	0.75 (0.66-0.79)	0.77 (0.69-0.81)	0.77 (0.69-0.85)	0.69 (0.54-0.77)	0.75 (0.69-0.81)
PEMAT actionability	0.00 (0.00-0.40)	0.40 (0.00-0.60)	0.40 (0.00-0.70)	0.00 (0.00-0.40)	0.00 (0.00-0.60)

^a^PEMAT: Patient Education Materials Assessment Tool.

### HON Code

The HON code certification was present on 12% (9/78) of the included website domains. Per category, there were 0% (0/14) HON-certified website domains in the nonacademic, 4% (1/28) in the academic, 24% (5/21) in the not-for-profit, and 20% (3/15) in the for-profit categories. No significant association was found between website categories and the presence of HON codes.

Websites with a HON code certification had higher total JAMA scores (*P*=.001) but did not have a significantly higher total DISCERN score, TACE content, FRES, FKGL, or PEMAT score.

### JAMA Score

The median JAMA score was 1.33 (IQR 0.83-1.75; Table 1). Information about authorship was absent on 88% (71/81) of the websites (Table 2). Of 81 websites, 8 (10%) mentioned author qualifications, 7 (9%) of which were authored or coauthored by a medical doctor. Sources of information were provided by 17% (14/81) of the websites (range 1-14 references). The date of publication of the latest update was mentioned in 42% (34/81) of websites. The median years since publication and update were 5.50 (IQR 2.25-9.75) and 1.50 (IQR 0.25-2.75), respectively. Only 11% (9/81) of the websites provided full disclosure.

Not-for-profit websites had significantly higher total JAMA scores than all other categories (*P*=.001 for nonacademic hospitals, *P*=.008 for academic hospitals, and *P*=.04 for for-profit organizations; Table 1). Not-for-profit websites were significantly associated with the presence of full disclosure and full attribution (*P*=.003 and *P*=.06, respectively).

There was a positive correlation between the total JAMA score and total DISCERN score (*r*_s_=+0.579; *P*<.001), total TACE content score (*r*_s_=+0.344; *P*=.002), FRES (*r*_s_=+0.356; *P*=.002), and FKGL (*r*_s_=−0.315; *P*=.006).

**Table 2:** Performance of websites on subdivisions of Journal of the American Medical Association benchmarks per category.

Aspects of benchmark disclosed on the website			Nonacademic hospitals (n=15), n (%)		Academic hospitals (n=29), n (%)		Not-for-profit organizations (n=21), n (%)		For-profit organizations (n=16), n (%)		Total (N=81), n (%)
**Authorship**											
	Name of author	0 (0)		2 (7)		4 (19)		3 (19)		9 (11)	
	Credentials of author	1 (7)		2 (7)		3 (14)		2 (13)		8 (10)	
	Affiliations of author	1 (7)		1 (3)		2 (10)		2 (13)		6 (7)	
	Adherence to all aspects of benchmark	0 (0)		1 (3)		1 (5)		2 (13)		4 (5)	
**Attribution**											
	References	0 (0)		2 (7)		8 (38)		4 (25)		14 (17)	
	Copyright information	15 (100)		28 (97)		18 (86)		15 (94)		76 (94)	
	Adherence to all aspects of benchmark	0 (0)		2 (7)		7 (33)		4 (25)		13 (16)	
**Currency**											
	Date created	3 (20)		7 (24)		9 (43)		2 (13)		21 (26)	
	Date updated	0 (0)		8 (28)		12 (57)		2 (13)		22 (27)	
	Adherence to all aspects of benchmark	0 (0)		5 (17)		3 (14)		1 (6)		9 (11)	
**Disclosure**											
	Site ownership	15 (100)		29 (100)		21 (100)		16 (100)		81 (100)	
	Sponsorship, advertising, underwriting, commercial funding arrangements, or support	7 (46.7)		11 (37.9)		11 (52)		7 (44)		36 (44)	
	Conflicts of interest	0 (0)		1 (3)		8 (38)		3 (19)		12 (15)	
	Adherence to all aspects of benchmark	0 (0)		0 (0)		6 (29)		3 (19)		9 (11)	

### DISCERN Score

The median DISCERN score was 47 (IQR 40.5-54.0), corresponding to fair quality (Table 1). No website had a total DISCERN score in the *excellent* range. The median score in the *Overall quality* section was 3.

**Figure 2:**
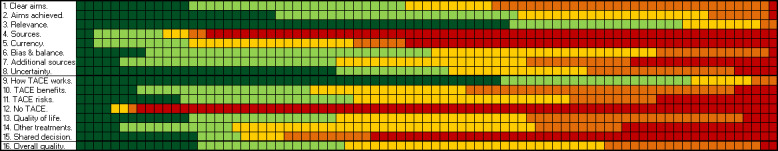
Distribution of scores on each component of the DISCERN score. Scores range from 1 (definitive no, red) to 5 (definitive yes, dark green). TACE: transarterial chemoembolization.

The questions with the lowest scores were *No TACE* and *Sources*, whereas *How TACE works* and *Relevance to patients* were the questions with the highest scores (Figure 2). The median score in the *Bias and balance* section was 4. Mention of benefits did not receive a more favorable scoring compared with risks, and vice versa (mean rank 30.48 vs 32.29; *P*=.27). No additional resources for further reading were provided on 21% (17/81) of the websites. The median score in the *Shared decision making* section was 1.

Not-for-profit websites had significantly higher total DISCERN scores than all other website categories (*P*=.006 for nonacademic hospitals, *P*=.03 for academic hospitals, and *P*<.001 for for-profit organizations; Table 1). They also scored higher in certain subdivisions (*Currency*, *Bias and balance*, *Reference to uncertainty*, and *Risks*) compared with all other categories (*P*<.05), and in *How TACE works* and in *Overall quality* compared with for-profit websites (*P*<.05).

Higher DISCERN scores were associated with higher TACE content scores (*r*=+0.701; *P*<.001).

### TACE Content Score

The median TACE content score was 35 (IQR 27-43; Table 1). Of 81 websites, only 4 (5%) websites reached a completeness of ≥70%, whereas only 1 (1%) website reached 90% completeness.

**Figure 3:**
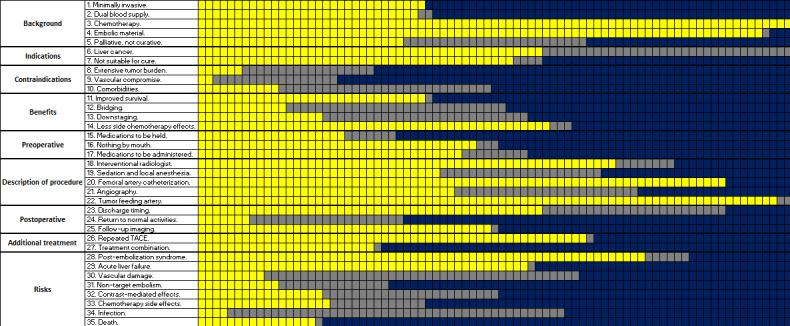
Distribution of scores on each component of the transarterial chemoembolization–content score. Scores range from 0 (no mention, blue) to 2 (full mention, yellow). TACE: transarterial chemoembolization.

Nearly all (78/81, 96%) websites mentioned the involvement of both chemotherapeutic and embolic agents in the procedure (Figure 3). Of 81 websites, 24 (30%) did not mention that the procedure is performed by an interventional radiologist; 45 (56%) websites failed to mention that the procedure involves exposure to x-rays along with injection of a contrast agent; 34 (42%) websites did not mention that TACE is offered when tumors are not amenable to curative treatments; 36 (45%) websites did not mention the nonchemotherapeutic medications that patients receive perioperatively; and 54 (67%) websites did not mention that certain medications need to be held before the procedure. The most underrepresented section was *Contraindications*, as 37% (30/81) of the websites failed to mention a single contraindication.

No benefits were mentioned on 5% (4/81) of the websites. Of 81 websites, 43 (53%) mentioned one or two benefits, whereas 34 (42%) websites mentioned two or more benefits. The most frequently mentioned benefit was “*less chemotherapy side effects*” (51/81, 63%), whereas the least commonly mentioned benefit was “*bridging to liver transplantation*” (42/81, 52%).

No risks were mentioned on 11% (9/81) of the websites. Of 81 websites, 14 (17%) mentioned one or two risks, whereas 58 (72%) mentioned three or more risks. The most commonly mentioned risks were “*postembolization syndrome*” (mentioned on 67/81, 83% of websites) and *“liver dysfunction*” (mentioned on 46/81, 57% of websites), whereas the least commonly mentioned risks were “*postoperative death*” (not mentioned on 64/81, 79% of websites) and “*nontarget embolism*” (not mentioned on 55/81, 68% of websites).

Not-for-profit websites had higher total TACE content scores than academic and for-profit websites (*P*=.04 and *P*=.001, respectively; Table 1). They also had significantly higher scores in *Risks* compared with all other categories (*P*=.01 for nonacademic hospitals, *P*<.001 for academic hospitals, and *P*<.001 for for-profit organizations) and in *Procedure description* compared with for-profit websites (*P*=.001). For-profit websites had a significantly lower score in *pre- and postprocedure considerations* compared with academic (*P*=.04) and not-for-profit websites (*P*=.009). No statistically significant difference existed between website categories in terms of *background*, *indications*, *contraindications*, *benefits*, and *additional treatments*.

### Flesch Reading Ease Score and Flesch-Kincaid Grade Level

The median FRES was 47.0 (IQR 38.7-57.8), and the median FKGL was 11.2 (IQR 8.9-12.6), corresponding to difficult degrees of readability (Table 1). Of the 76 websites, only 2 (3%) had a readability level of 7th grade, whereas 0 (0%) websites were within the recommended readability level of 6th grade or lower (Table 3). Moreover, 0 (0%) websites had an FRES corresponding to the *easy* or *very easy* readability level. Most websites (48/76, 63%) were deemed *difficult* or *very difficult* to read.

No significant difference was found in the FRES and FKGL between the website categories (Table 1). Websites with a higher FRES were associated with higher total DISCERN scores (*r*=+0.411; *P*<.001) and total TACE content scores (*r*=+0.250; *P*=.03). Websites with a lower FKGL were associated with higher total DISCERN scores (*r*=−0.392; *P*<.001) and total TACE content scores (*r*=−0.289; *P*=.01).

**Table 3:** Percentage of websites per each Flesch Reading Ease Scale category and corresponding Flesch-Kincaid Grade Level.

Flesch Reading Ease Scale	US Department of Health and Human Sciences writing style difficulty	Corresponding grade	Number of websites (n=76), n (%)	Corresponding Flesch-Kincaid Grade Level, median (IQR)
91-100	Very easy	5th	0 (0)	N/A^a^
81-90	Easy	6th	0 (0)	N/A
71-80	Fairly easy	7th	2 (3)	6.7 (6.4-7.0)
61-70	Standard	8th-9th	13 (17)	8.0 (7.4-8.5)
51-60	Fairly difficult	10th-12th	13 (17)	9.2 (8.8-10.6)
31-50	Difficult	College student	37 (49)	11.7 (11.0-12.6)
0-30	Very difficult	College graduate	11 (15)	15.1 (14.4-16.3)

^a^N/A: not applicable.

### Patient Education Materials Assessment Tool

The median understandability score was 0.75 (IQR 0.69-0.81; Table 1). Of 81 websites, 49 (61%) scored higher than 70% on the understandability score; 11 (14%) websites had distracting content; and 17 (21%) websites used medical vocabulary without adequate explanation (Table 4). Of the 36 websites that used visual aids (images or videos), 17 (47%) were deemed useful and 18 (50%) displayed captions.

The median actionability score was 0.0 (IQR 0.0-0.6; Table 1). Of 81 websites, 13 (16%) websites scored higher than 70% on the actionability score; 42 (52%) websites scored zero; 39 (48%) websites provided at least one action that consumers should take with regards to TACE (eg, holding certain home medications before the procedure, refraining from certain activities after the procedure, and discussing their concerns with their doctor; Table 4).

There were no significant correlations between the website category and PEMAT performance (Table 1). There was a positive correlation between PEMAT understandability and total DISCERN scores (*r*_s_=+0.271; *P*=.02). There was a positive correlation between PEMAT actionability and total DISCERN score (*r*_s_=+0.336; *P*=.002) and total TACE content score (*r*_s_=+0.376; *P*=.001). There was a positive correlation between PEMAT understandability or actionability scores and the FRES (*r*_s_=+0.372, *P*=.001 and *r*_s_=+0.704, *P*<.001, respectively) and a negative correlation between PEMAT understandability or actionability scores and the FKGL (*r*_s_=−0.346, *P*=.002 and *r*_s_=−0.695, *P*<.001, respectively).

**Table 4:** Patient Education Materials Assessment Tool for understandability and actionability and performance of websites per website category.

Patient Education Materials Assessment Tool items				Nonacademic hospitals				Academic hospitals				Not-for-profit organizations						For-profit organizations									Total			
				n (%)		Total, n		n (%)		Total, n		n (%)				Total, n		n (%)					Total, n			n (%)				Total, n
**Understandability**																														
	**Content**																													
		The material makes its purpose completely evident.	14 (93)		15		29 (100)		29		20 (95)				21		16 (100)					16			79 (98)				81	
		The material does not include information or content that distracts from its purpose.	13 (87)		15		28 (97)		29		19 (91)				21		10 (63)					16			70 (86)				81	
		The material uses common, everyday language.	15 (100)		15		25 (86)		29		19 (91)				21		13 (81)					16			72 (90)				81	
		Medical terms are used only to familiarize the audience with the terms.	10 (67)		15		21 (72)		29		20 (95)				21		13 (81)					16			64 (79)				81	
		The material uses active voice.	8 (53)		15		19 (66)		29		9 (43)				21		7 (44)					16			43 (53)				81	
	**Use of numbers**																													
		Numbers appearing in the material are clear and easy to understand.	9 (100)		9		22 (100)		22				21 (100)		21				12 (100)		12			64 (100)				64		
		The material does not expect the user to perform calculations.	15 (100)		15		29 (100)		29				20 (95)		21				15 (94)		16			79 (98)				81		
	**Organization**																													
		The material breaks or *chunks* information into short sections.	15 (100)		15		26 (90)		29				20 (95)		21				14 (88)		16			75 (93)				81		
		The material’s sections have informative headers.	15 (100)		15		26 (93)		29				21 (100)		21				12 (75)		16			74 (91)				81		
		The material presents information in a logical sequence.	14 (93)		15		23 (79)		29				19 (91)		21				8 (50)		16			64 (79)				81		
		The material provides a summary.	0 (0)		15		0 (0)		29				1 (5)		21				0 (0)		16			1 (1)				81		
	**Layout and design**																													
		The material uses visual cues to draw attention to key points.	13 (80)		15		21 (76)		29				16 (76)		21				9 (56)		16			59 (73)				81		
		Text on the screen is easy to read (*for audiovisual content*).	N/A^a^		N/A		1 (100)		1				N/A		N/A				2 (100)		2			3 (100)				3		
		The material allows the user to hear the words clearly (*for audiovisual content*).	1 (100)		1		1 (100)		1				N/A		N/A				1 (50)		2			3 (75)				4		
	**Use of visual aids**																													
		The material uses visual aids whenever they could make content more easily understood.	1 (7)		15		6 (21)		29				3 (14)		21				6 (38)		16			16 (20)				81		
		The material’s visual aids reinforce rather than distract from the content.	1 (20)		5		6 (46)		13				4 (50)		8				6 (60)		10			17 (47)				36		
		The material’s visual aids have clear titles or captions.	0 (0)		5		7 (54)		13				5 (63)		8				6 (60)		10			18 (50)				36		
		The material uses illustrations and photographs that are clear and uncluttered.	3 (60)		5		12 (92)		13				8 (100)		8				8 (80)		10			31 (86)				36		
		The material uses simple tables with short and clear row and column headings.	N/A		N/A		N/A		N/A				N/A		N/A				N/A		N/A			N/A				N/A		
**Actionability**																														
	The material clearly identifies at least one action the user can take.			5 (33)		15		18 (62)		29				11 (52)		21				5 (31)		16			39 (48)				81	
	The material addresses the user directly when describing actions.			5 (33)		15		18 (62)		29				11 (52)		21				5 (31)		16			39 (48)				81	
	The material breaks down any action into manageable, explicit steps.			1 (7)		15		11 (38)		29				7 (33)		21				2 (13)		16			21 (26)				81	
	The material provides a tangible tool whenever it could help the user take action.			0 (0)		15		6 (21)		29				5 (24)		21				2 (13)		16			13 (16)				81	
	The material provides simple instructions or examples of how to perform calculations.			N/A		N/A		N/A		N/A				N/A		N/A				N/A		N/A			N/A				N/A	
	The material explains how to use charts, graphs, tables, or diagrams to take actions.			N/A		N/A		N/A		N/A				N/A		N/A				N/A		N/A			N/A				N/A	
	The material uses visual aids whenever they could make it easier to act on the instructions.			0 (0)		15		0 (0)		29				0 (0)		21				1 (6)		16			1 (1)				81	

^a^N/A: not applicable.

## Discussion

### Principal Findings

TACE is a valuable treatment option for select cases of primary or metastatic liver tumors. As such, it will remain a reason for thousands of web searches by patients with liver cancer and their families. Despite the multitude of available websites, no previous study has explored the reliability, quality, readability, understandability, actionability, and completeness of websites providing consumer-directed information on TACE. Our systematic review demonstrates that these websites are generally unreliable and are characterized by fair quality, insufficient content, adequate understandability, and poor readability and actionability.

There is no consensus regarding the optimal method for rating health-related websites. To date, there has been considerable heterogeneity among studies in terms of search engines or keywords used, number of screened websites, inclusion criteria, parameters evaluated, and assessment tools. Some studies focus only on one parameter (eg, readability) [21,39-41], whereas others address quality and content as well [23,24,28,42-44]. Multiple quality and readability assessment tools exist, the selection of which relies on the discretion of the study group. On the contrary, the assessment of the content provided by websites is topic-specific and requires a scoring tool dedicated to the topic of interest.

Many studies have generated *topic-specific scores* to evaluate the accuracy and completeness of websites [28,42-44]. In this study, we generated a novel TACE-specific score that includes 35 key points, which we believe should be covered by any website that aspires to adequately educate patients on TACE. Our results showed that, on average, websites had 50% completeness, indicating a significant lack of content. Although the procedure was adequately described, certain benefits and risks were missed by many websites, and contraindications were largely neglected. One striking finding of our study is that almost 30% of websites failed to mention that the procedure was performed by an interventional radiologist. Of note, one website stated that the procedure was performed by *a technician*. Given the challenge of raising public awareness that IR has been facing, not only in the general public but also in the oncology community, emphasizing the performing specialty is of utmost importance [18,45].

Another important finding of our study is the strikingly poor reporting of authorship, currency, and references in the included websites, irrespective of their category. Mention of these features is essential for any health-related website that aspires to provide credible information and gain the trust of readers [14]. Moreover, 28% (22/78) of websites did not provide a medical disclaimer. A medical disclaimer would remind readers that the accuracy of the content provided is not guaranteed and that direct patient-physician discussion is irreplaceable. A study of 512 participants showed that 60% of people believe that the information they find on the internet is the *same as or better than* the information provided by their doctors [46]. Therefore, a medical disclaimer would highlight that health decision-making cannot be shaped solely based on self-education and that consultation with a medical professional is essential. Ideally, disclaimers should be readily visible on the same page as the medical content, instead of an obscure spot at the bottom of a website, as in most websites we evaluated. Readers are very unlikely to specifically search for disclosure statements [47].

The lack of these reliability parameters is also reflected by the low percentage (9/78, 12%) of websites that carried the HON certification. As a simple identifier of website objectivity and transparency, the HON code badge directs internet users toward more reliable websites. Our results showed that websites carrying a HON badge had higher JAMA scores, which is expected because these 2 indices share certain similar parameters. However, the presence of HON certification was *not* associated with more favorable scores on the quality, completeness, and readability tools that we used. This proves that these websites may be trustworthy but may not adequately describe the health-related topic or may do so but in a way that is not reader friendly. Therefore, the HON badge should not be perceived as the sole identifier of high-quality websites.

There are dozens of readability formulas available for quantitative readability assessment [40]. Generally, their main presumption is that longer sentences and longer words are more difficult to read. Although this may hold true, it does not consider the coherence of the text or the literal complexity of the words. For instance, a short word would be considered easy to read by the formulas, but it may be too sophisticated for the average reader. Moreover, readability results may vary based on word sampling, text formatting, and calculation tools [48].

To avoid a one-sided approach to readability, we chose to evaluate websites using PEMAT as an additional tool. PEMAT is not a formula-based readability index but a subjective scoring system dedicated to health-related materials. It evaluates the website holistically, taking into consideration not only the text but also the organization of the information, the effectiveness of the multimedia, and the presence of distracting content. These factors determine how likely the website is to engage readers and hold their attention.

The results from our readability assessment showed that the median readability level of the websites was at the 11th grade level, well above the recommended 6th grade threshold. This indicates that patients with low health literacy are at a disadvantage, as they would not be able to comprehend the web-based resources available for TACE. Although 61% (49/81) of websites were deemed understandable, 21% (17/81) of the websites used medical terms without adequate explanation, and 14% (11/81) of the websites had distracting content. Moreover, approximately half of the websites did not mention a single action that readers must take. Suggesting clear actions would enable readers to make informed decisions about their care and therefore improve their health literacy [49,50].

Websites with a higher word count scored better on the TACE content and PEMAT actionability scores. This is expected, as more words tend to provide more content and therefore more complete information. However, websites need to find the right *balance* between providing adequate content and maintaining optimal readability. Our results showed that websites with a higher word count were *not* significantly associated with higher PEMAT understandability scores. Therefore, longer texts need to provide comprehensible information without distracting the reader.

The websites we evaluated seemed to underestimate the value of multimedia, as 56% (45/81) of websites did not use a single illustration or video. Of the websites that provided visual aids, only half were considered to be reinforcing or adequately captioned. Visual aids deliver information in a way that is more familiar to some patients and do not require a high level of literacy [51]. Given that TACE is a procedure unfamiliar to the general public, the use of multimedia could be helpful in describing the process in a simplified manner. Spoken animation has been found to be the most efficient way of communicating complex health information to people with low health literacy and could prove useful in the context of TACE [52].

Not-for-profit websites were found to provide the most reliable, high-quality, and complete content compared with all other website categories, as reflected by the significantly higher scores in JAMA, DISCERN, and TACE- content scores. They also mentioned more risks and were deemed less biased. This is in line with other studies that have found more favorable quality scores on nonprofit websites [22,43,53-55]. Nonprofit organizations aspire to educate the public in an objective and balanced way without seeking direct financial benefits, as opposed to hospitals and companies. As such, they appear to be trustworthy sources of patient information.

### Recommendations for Website Developers

When creating a website that aspires to provide health education, content creators need to consult medical professionals with expertise on the desired topic. The name and credentials of the author, the date of creation and of last update, and the references should by no means be neglected so that the reader is ascertained that the information is credible and reliable. A medical disclaimer should be considered an essential feature and should be clearly and distinctly located on the webpage.

There are multiple ways to enhance the learning process of website users. Illustrations and animated videos remarkably increase the understandability of the presented content and therefore should be more broadly used. Summary tables may draw attention to take-away points in a simplified way and aid in the decision-making process. Brief interactive quizzes at the end of the article could also consolidate the reader’s knowledge. The presence of resources for additional reading is helpful in directing patients to other useful websites with pertinent information.

An average web user does not have the capacity to screen websites and proceed to those that address consumers. A useful addition to search engines would be to create a web browser extension that would provide a sign next to each health-related website (similar to the HON code badge), stating whether it is appropriate for consumer education. Another option for websites would be to either have two separate versions, one for consumers and one for professionals (eg, UpToDate [56] and Merck manual [57]), or provide plain-language summaries (eg, Cochrane evidence [58]). These suggestions would enable patients to be readily directed to websites that provide information in the lay language.

### Limitations

Our study has several limitations. Quality assessment tools may introduce a subjective bias; however, a considerable attempt was made to standardize the grading process. Discrepancies were resolved by discussion, and no interrater variability was measured. No blinding existed between the reviewers and the website owner. Furthermore, a new scoring system for TACE has been suggested, which has not been validated by other studies. Website rank on the search engines was not documented, as websites may appear on different ranks depending on the search history and location of each user. Content provided by supplementary videos (when available) was considered; however, websites providing only video content and foreign-language websites were excluded. Studies assessing such websites could shed further light on the quality of existing resources. Finally, websites are dynamic and may have been updated by the time the quality assessment took place.

### Conclusions

To our knowledge, this is the first study to evaluate web-based resources that provide information about TACE to patients. Our comprehensive assessment showed that the materials currently available on the web are unreliable, difficult to read, easy to understand but difficult to act upon, and do not provide complete information about TACE. Websites were characterized by fair quality and did not meet the standards for shared decision-making. Website developers are encouraged to revise their content and provide transparent, complete, and readable resources so that patients can make informed and safe decisions. Certain suggestions are made that could help high-quality and reader-friendly websites become more accessible to consumers.

## Appendix

Multimedia Appendix 1 DISCERN score.

Multimedia Appendix 2 Transarterial chemoembolization content score.

Multimedia Appendix 3 List of included websites.

## Abbreviations

FKGL: Flesch-Kincaid Grade Level
FRES: Flesch Reading Ease Score
HON: Health On the Net
IR: interventional radiology
JAMA: Journal of the American Medical Association
PEMAT: Patient Education Materials Assessment Tool
TACE: transarterial chemoembolization
