# Supplementary material for: The Value of Web-Based Patient Education Materials on Transarterial Chemoembolization: Systematic Review
Source: JMIR Cancer. 2021 May 7;7(2):e25357. doi: 10.2196/25357 (PMC8140383; doi:10.2196/25357)
Supplement: Multimedia Appendix 1 [file cancer_v7i2e25357_app1.docx]

**Multimedia Appendix 1: DISCERN score.**

|  | **RELIABILITY** |
| --- | --- |
| 1 | Are the aims clear? |
| 2 | Does it achieve its aims? |
| 3 | Is it relevant? |
| 4 | Is it clear what sources of information were used to compile the publication? |
| 5 | Is it clear when the information used or reported in the publication was produced? |
| 6 | Is it balanced and unbiased? |
| 7 | Does it provide details of additional sources of support and information? |
| 8 | Does it refer to areas of uncertainty? |
|  | **QUALITY** |
| 9 | Does it describe how TACE works? |
| 10 | Does it describe the benefits of TACE? |
| 11 | Does it describe the risks of TACE? |
| 12 | Does it describe what would happen if TACE is not used? |
| 13 | Does it describe how TACE affects overall quality of life? |
| 14 | Is it clear that there may be possible treatment choices other than TACE? |
| 15 | Does it provide support for shared decision-making? |
|  | **OVERALL RATING** |
| 16 | What is the overall quality of the publication as a source of information about TACE? |
