# Supplementary material for: The Value of Web-Based Patient Education Materials on Transarterial Chemoembolization: Systematic Review
Source: JMIR Cancer. 2021 May 7;7(2):e25357. doi: 10.2196/25357 (PMC8140383; doi:10.2196/25357)
Supplement: Multimedia Appendix 2 [file cancer_v7i2e25357_app2.docx]

**Multimedia Appendix 2: TACE-content score.**

|  | **BACKGROUND** |
| --- | --- |
| 1 | TACE is a minimally-invasive procedure. |
| 2 | TACE takes advantage of the dual blood supply of the liver (hepatic artery and portal vein). |
| 3 | TACE involves injection of chemotherapy locally into the tumor. |
| 4 | TACE also involves injection of embolic material that cuts the blood supply of the tumor. |
| 5 | TACE is a palliative, not curative, treatment option. |
|  | **INDICATIONS** |
| 6 | TACE is used to treat primary or metastatic liver cancer. |
| 7 | TACE is used to treat tumors that are not suitable for curative treatments (resection, liver transplantation, radiofrequency/ microwave ablation). |
|  |  |
|  | **CONTRAINDICATIONS** |
| 8 | Extensive tumor burden: within the liver or extrahepatic. |
| 9 | Vascular compromise: portal vein thrombosis, vascular invasion, poor hepatic arterial flow. |
| 10 | Comorbidities: decompensated liver function, biliary obstruction, renal failure, coagulopathy. |
|  | **BENEFITS** |
| 11 | Improved survival compared to supportive care. |
| 12 | TACE can halt tumor growth while a patient is on a waiting list for a liver transplant (bridging). |
| 13 | TACE can decrease the size of the tumor and make it amenable for liver transplantation (downstaging). |
| 14 | TACE has less side effects compared to systemic chemotherapy. |
|  | **PRE-OPERATIVE CONSIDERATIONS** |
| 15 | Certain medications (e.g. blood thinners, metformin) may need to be held prior to and/or after the procedure. |
| 16 | The patient should not eat solid foods 6 hours prior to the procedure. |
| 17 | The patient will be given painkillers, anti-nausea, anti-allergy medications and possibly antibiotics. |
|  | **DESCRIPTION OF PROCEDURE** |
| 18 | TACE is performed by an interventional radiologist. |
| 19 | The procedure is performed under conscious sedation and local anesthesia. |
| 20 | Vascular access is achieved by catheterizing the femoral artery in the groin area or the radial artery at the wrist. |
| 21 | Contrast material is injected to visualize the blood vessels with the use of x-rays. |
| 22 | A catheter is advanced into the tumor feeding artery where the chemoembolic agents are administered. |
|  | **POST-OPERATIVE CONSIDERATIONS** |
| 23 | After a bed rest of about 6 hours, the patient may be discharged the same or next day. |
| 24 | Certain activities need to be restricted for 1 week following the procedure. |
| 25 | Follow-up imaging will be needed for response monitoring. |
|  | **ADDITIONAL TREATMENT** |
| 26 | Repeated TACE sessions are often needed. |
| 27 | TACE can be combined with other treatments (e.g. thermal ablation, radiation, chemotherapy). |
|  | **RISKS** |
| 28 | Post- embolization syndrome (fever, nausea, vomiting, fatigue, abdominal pain). |
| 29 | Deterioration of liver function. |
| 30 | Vascular damage: access site bleeding/ pseudoaneurysm, hepatic artery dissection/thrombosis. |
| 31 | Non- target embolism: liver infarction, cholecystitis, GI ulceration, oil embolism, pulmonary embolism. |
| 32 | Contrast- mediated side effects: allergy, acute kidney injury. |
| 33 | Chemotherapy side effects (e.g. bone marrow suppression). |
| 34 | Infection: liver abscess, bloodstream infection. |
| 35 | Death. |
