# Supplementary material for: The Value of Web-Based Patient Education Materials on Transarterial Chemoembolization: Systematic Review
Source: JMIR Cancer. 2021 May 7;7(2):e25357. doi: 10.2196/25357 (PMC8140383; doi:10.2196/25357)
Supplement: Multimedia Appendix 3 [file cancer_v7i2e25357_app3.docx]

**Multimedia Appendix 3: List of included websites.**

|  | **URL** | **WEBSITE OWNER** |
| --- | --- | --- |
|  | **NON- ACADEMIC HEALTHCARE INSTITUTIONS** |  |
| 1 | <https://www.azuravascularcare.com/medical-services/interventional-oncology-treatments/chemoembolization> | Fresenius Medical Care |
| 2 | <https://www.centracare.com/services/imaging-services/patient-instructions/chemoembolization> | Centra Care |
| 3 | <https://www.chomp.org/services/radiology/interventional-radiology/chemoembolization> | Montage Health |
| 4 | <https://www.gwinnettmedicalcenter.org/services/imaging/interventional-radiology/chemoemolization> | Northside Hospital |
| 5 | <https://www.hamiltonhealthsciences.ca/transcatheter-arterial-chemoembolization-tace-deciding-to-have-a-tace-procedure> | Hamilton Health Sciences |
| 6 | <https://www.hcahealthcare.co.uk/our-services/treatments/chemoembolisation-tace> | HCA Healthcare UK |
| 7 | <https://www.intracare.co.nz/Procedures-Treatments/Interventional-Oncology/Targeted-cancer-therapies/Transcatheter-Arterial-Chemoembolisation-TACE> | Intra |
| 8 | <http://www.irtreatment.org/procedures-and-treatments/vascular/chemoembolization-tace-for-liver-cancer.html> | Interventional Radiology, Kovai Medical Center and Hospital |
| 9 | <https://www.medstarhealth.org/mhs/our-services/interventional-radiology/services/interventional-radiology-cancer/trans-arterial-chemoembolization-tace> | Medstar Health |
| 10 | <https://www.mercy.com/health-care-services/cancer-care-oncology/specialties/liver-cancer-pancreatic-cancer/treatments/chemoembolization-for-liver-cancer> | Mercy Health |
| 11 | https://www.mercy.com/health-care-services/cancer-care-oncology/specialties/liver-cancer-pancreatic-cancer/treatments/pancreas-chemoembolization |  |
| 12 | <https://www.phyathai.com/article_detail/2162/en/Transarterial_ChemoEmbolization_(TACE)_for_Hepatocellular_Carcinoma> | Phyathai Hospital |
| 13 | <https://www.rochesterregional.org/services/imaging-radiology/diagnostic-imaging-rochester-general/interventional-radiology/chemoembolization> | Rochester Regional Health |
| 14 | <https://www.roswellpark.org/cancer/neuroendocrine-carcinoid/treatment/non-surgical-techniques/chemoembolization> | Roswell Park Cancer Institute |
| 15 | <http://www.union.org/new/consent_form_files/MEI-01e.pdf> | Union Medical Centre |
|  | **ACADEMIC HEALTHCARE INSTITUTIONS** |  |
| 16 | <https://www.cedars-sinai.edu/Patients/Programs-and-Services/Imaging-Center/For-Patients/Exams-by-Procedure/Interventional-Radiology/Chemoembolization---Liver.aspx> | Cedars-Sinai |
| 17 | <http://www.radiology-berlin.de/mitt-de/en/home-en/liver/tace> | Charité Berlin |
| 18 | <https://www.cooperhealth.org/services/chemoembolization-liver-cancer> | Cooper University Health Care |
| 19 | <https://www.hopkinsmedicine.org/interventional-radiology/procedures/chemoembolization> | Johns Hopkins Medicine |
| 20 | <http://radiologie-uni-frankfurt.de/content/e6796/e13478/e13775/e16256/index_eng.html> | Institute for Diagnostic and Interventional Radiology |
| 21 | <https://northeast.jeffersonhealth.org/programs-and-services/radiology/interventional-radiology/chemoembolization> | Jefferson Health - Northeast |
| 22 | <https://www.massgeneral.org/interventional-radiology/treatments-and-services/tace> | The General Hospital Corporation |
| 23 | <https://www.leedsth.nhs.uk/assets/Uploads/0da75560c7/Trans-Arterial-Chemoembolisation-TACE.pdf> | The Leeds Teaching Hospitals NHS Trust |
| 24 | <http://www.plymouthhospitals.nhs.uk/download.cfm?doc=docm93jijm4n1014.doc&ver=1211> | University Hospitals Plymouth NHS Trust |
| 25 | [https://www.nm.org/conditions-and-care-areas/treatments/chemoembolization  https://www.nm.org/patients-and-visitors/patient-education/tests-and-procedure-information/chemoembolization-patient-education](https://www.nm.org/conditions-and-care-areas/treatments/chemoembolization) | Northwestern Medicine |
|  |  |  |
| 26 | [https://www.nm.org/patients-and-visitors/patient-education/tests-and-procedure-information [PDF selection]](https://www.nm.org/patients-and-visitors/patient-education/tests-and-procedure-information%20%5bPDF%20selection%5d) |  |
| 27 | <https://www.ntuh.gov.tw/cmio/Fpage.action?muid=2377&fid=2229> | National Taiwan University Hospital Healthcare System |
| 28 | <https://www.ohsu.edu/sites/default/files/2019-04/TACE-Handout.pdf> | Oregon Health & Science University |
| 29 | <http://publicdocuments.sth.nhs.uk/PIL4071.PDF> | Sheffield Teaching Hospitals NHS Foundation Trust |
| 30 | <https://www.semc.org/services-directory/imaging-radiology/interventional-radiology/chemoembolization> | Steward Health Care |
| 31 | <https://stanfordhealthcare.org/medical-treatments/c/chemoembolization.html> | Stanford Health Care |
| 32 | <https://ukhealthcare.uky.edu/transplant-center/services/additional-services/chemoembolization> | University of Kentucky |
| 33 | <https://www.rogelcancercenter.org/liver-cancer/transarterial-chemoembolization> | Rogel Cancer Center University of Michigan |
| 34 | <https://www.uabmedicine.org/documents/142028/233977/Transarterial+Chemoembolization+-+For+Patients/9337386b-e376-4329-9047-71820036317b?version=1.0> | University of Alabama Medicine |
| 35 | <https://www.uclh.nhs.uk/PandV/PIL/Patient%20information%20leaflets/DEB-TACE.pdf> | University College London Hospitals NHS Foundation Trust |
| 36 | <https://www.uclahealth.org/radiology/interventional-oncology/chemoembolization> | University of California Los Angeles Health |
| 37 | <https://transplant.surgery.ucsf.edu/conditions--procedures/hepatic-artery-embolization.aspx> | The Regents of the University of California |
| 38 | <https://www.uhn.ca/PatientsFamilies/Health_Information/Health_Topics/Documents/Transarterial_Chemoembolization_TACE.pdf> | University Health Network |
| 39 | <https://www.pennmedicine.org/for-patients-and-visitors/find-a-program-or-service/interventional-radiology/chemoembolization> | Trustees of the University of Pennsylvania |
| 40 | <https://www.upmc.com/services/liver-cancer/treatments/intrahepatic-chemotherapy> | University of Pittsburgh Medical Center |
| 41 | <https://utswmed.org/conditions-treatments/chemoembolization> | The University of Texas Southwestern Medical Center |
| 42 | <https://healthonline.washington.edu/sites/default/files/record_pdfs/IR-Chemoembolization.pdf> | University of Washington |
| 43 | <https://www.uwhealth.org/healthfacts/radiology-invasive/5763.pdf> | University of Wisconsin Hospitals and Clinics Authority |
| 44 | http://healthlibrary.vanderbilthealth.com/Search/3,85501 | Vanderbilt University Medical Center |
|  | **NOT-FOR-PROFIT ORGANIZATIONS** |  |
| 45 | <http://www.aldrodriguezliverfoundation.com/transarterial-chemoembolization-tace-for-liver-cancer> | Al D. Rodriguez Liver Foundation |
| 46 | <https://www.cancer.org/cancer/liver-cancer/treating/embolization-therapy.html> | American Cancer Society |
| 47 | <https://www.bsir.org/media/resources/BSIR_Patient_Leaflet_-_Transarterial_Chemoembolisation_TACE.pdf> | British Society of Interventional Radiology |
| 48 | <https://www.cancer.ca/en/cancer-information/cancer-type/liver/treatment/transarterial-chemoembolization/?region=on> | Canadian Cancer Society |
| 49 | <https://www.cancerresearchuk.org/about-cancer/liver-cancer/treatment/chemoembolisation> | Cancer Research UK |
| 50 | <https://www.cirse.org/patients/ir-procedures/embolisation-in-oncology/> | Cardiovascular and Interventional Radiological Society of Europe |
| 51 | [https://www.healthwise.net/osumychart/find/search.aspx?searchTerm=tace https://www.healthwise.net/osumychart/Content/StdDocument.aspx?DOCHWID=custom.jc0454](https://www.healthwise.net/osumychart/find/search.aspx?searchTerm=tace) | Healthwise Inc. |
|  |  |  |
| 52 | <http://www.hksir.org.hk/document/PatInfLeaflet/Eng/pdf/EN38%20TACE%20eng%202010.pdf> | Hong Kong Society of Interventional Radiology |
| 53 | <http://www21.ha.org.hk/smartpatient/SPW/static/migration/files/PDF/QM/SRG-21.pdf> | Hospital Authority |
| 54 | <https://www.insideradiology.com.au/transarterial-chemoembolisation> | The Royal Australian and New Zealand College of Radiologists |
| 55 | <https://jamanetwork.com/journals/jamaoncology/fullarticle/2457397> | American Medical Association |
| 56 | <http://library.nhsggc.org.uk/mediaAssets/Diagnostic%20Imaging/254363%20-%20TACE%20Procedure%20(Transarterial%20Chemoembolisation).pdf> | NHS Greater Glasgow And Clyde |
| 57 | <https://www.scan.scot.nhs.uk/Documents/Chemoembolisation%20(TACE).pdf> | NHS Lothian |
| 58 | <http://www.wales.nhs.uk/sitesplus/866/opendoc/156695> | Public Health Wales NHS Trust |
| 59 | <http://www.northflorida.va.gov/NORTHFLORIDA/patients/education/Chemoembolization.doc> | North Florida/South Georgia Veterans Health System |
| 60 | <https://www.oncolink.org/cancer-treatment/procedures-diagnostic-tests/interventional-radiology-procedures/chemoembolization> | Trustees of the University of Pennsylvania |
| 61 | <https://www.health.qld.gov.au/__data/assets/pdf_file/0022/153715/medical_imaging_32.pdf> | Queensland Government |
| 62 | <https://www.radiologyinfo.org/en/info.cfm?pg=chemoembol> | Radiological Society of North America |
| 63 | <https://www.rdehospital.nhs.uk/documents/patient-information-leaflets/radiology/patient-information-leaflet-transarterial-chemoembolisation-tace-for-liver-tumours.pdf> | Royal Devon and Exeter NHS Foundation Trust |
| 64 | <https://www.hepatitis.va.gov/liver-cancer/treatment/index.asp> | US Department of Veteran Affairs |
| 65 | <https://en.wikipedia.org/wiki/Transcatheter_arterial_chemoembolization> | Wikimedia Foundation, Inc. |
|  | **FOR-PROFIT ORGANIZATIONS** |  |
| 66 | <https://www.albanyir.com/chemoembolization> | Albany IR |
| 67 | <http://www.gep-net.com/english%20page/mainpage/subpage/TACE_EGEP.htm> | Center for Neuroendocrine Tumors |
| 68 | <https://www.drrogan.com/patient-information/transarterial-chemoembolisation-for-liver-cancer/> | Dr Chris Rogan |
| 69 | [https://www.drugs.com/cg/chemoembolization-cancer-therapy.html https://www.drugs.com/cg/chemoembolization-cancer-therapy-ambulatory-care.html https://www.drugs.com/cg/chemoembolization-cancer-therapy-discharge-care.html](https://www.drugs.com/cg/chemoembolization-cancer-therapy-ambulatory-care.html) | Drugs.Com |
|  |  |  |
|  |  |  |
| 70 | <http://erabillings.com/services/transarterial-chemoembolization-tace> | Eastern Radiological Associates (ERA) |
| 71 | [http://www.flinterventionalspecialists.com/cancer-treatments/cancer-chemoembolization http://www.flinterventionalspecialists.com/media/pages/Chemoembolization_WTE_5-2016.pdf](http://www.flinterventionalspecialists.com/cancer-treatments/cancer-chemoembolization) | Florida Interventional Specialists |
|  |  |  |
| 72 | <https://ivcnorthwest.com/treatment/chemoembolization/> | Interventional and Vascular Consultants Northwest |
| 73 | <https://www.medicinenet.com/chemo_infusion_and_chemoembolization_of_liver/article.htm> | MedicineNet, Inc. |
| 74 | <http://www.hemonc101.com/What-is-chemoembolization-s/1862.htm> | Miami Hematology and Oncology Associates |
| 75 | <https://northsideradiology.com/procedures/chemoembolization> | Northside Radiology Associates |
| 76 | <http://www.rimarad.com/?page_id=417> | Renaissance Medical Imaging Associates |
| 77 | <https://www.siemens-healthineers.com/en-uk/clinical-specialities/oncology/interventional-oncology-procedures/transarterial-chemoembolization> | Siemens Healthcare Limited |
| 78 | <http://www.sir.net.au/TACE.html> | Sydney Interventional Radiology |
| 79 | <https://www.topdoctors.co.uk/medical-dictionary/chemoembolization> | Top Doctors UK |
| 80 | <https://www.topdoctors.co.uk/medical-articles/what-is-tace> |  |
| 81 | <https://www.wisegeek.com/what-is-transarterial-chemoembolization.htm> | WiseGEEK |
